# Molecular mechanism of flower colour formation in *Rhododendron simsii* Planchon revealed by integration of microRNAome and RNAomics

**DOI:** 10.1093/aobpla/plae053

**Published:** 2024-10-14

**Authors:** Jun Fu, Chuanchuan Tian, Xuchun Wan, Ruibin Hu, Jiaojun Yu, Jialiang Zhang, Shuzhen Wang

**Affiliations:** College of Biology and Agricultural Resources, Huanggang Normal University, Huanggang, Hubei Province, 438000, China; College of Biology and Agricultural Resources, Huanggang Normal University, Huanggang, Hubei Province, 438000, China; College of Biology and Agricultural Resources, Huanggang Normal University, Huanggang, Hubei Province, 438000, China; College of Biology and Agricultural Resources, Huanggang Normal University, Huanggang, Hubei Province, 438000, China; College of Biology and Agricultural Resources, Huanggang Normal University, Huanggang, Hubei Province, 438000, China; College of Biology and Agricultural Resources, Huanggang Normal University, Huanggang, Hubei Province, 438000, China; College of Biology and Agricultural Resources, Huanggang Normal University, Huanggang, Hubei Province, 438000, China

**Keywords:** Flower colour, gene expression profiling, miRNA–RNA regulatory network, molecular mechanism, *Rhododendron simsii* Planchon

## Abstract

Systems-wide understanding of gene expression profile regulating flower colour formation in *Rhododendron simsii* Planchon is insufficient. In this research, integration analysis of ribonucleic acid (RNA)omics and microRNAome were performed to reveal the molecular mechanism of flower colour formation in three *R. simsii* varieties with red, pink and crimson flowers, respectively. Totally, 3129, 5755 and 5295 differentially expressed gene (DEG)s were identified through comparative transcriptome analysis between ‘Red variety’ and ‘Pink variety’ (1507 up-regulated and 1622 down-regulated), ‘Red variety’ and ‘Crimson variety’ (2148 up-regulated 3607 down-regulated), as well as ‘Pink variety’ and ‘Crimson variety’ (2089 up-regulated and 3206 down-regulated), which were involved in processes of ‘catalytic activity’, ‘binding’, ‘metabolic process’ and ‘cellular process’, as well as pathways of ‘metabolic pathways’, ‘biosynthesis of secondary metabolites’, ‘plant-pathogen interaction’ and ‘phenylpropanoid biosynthesis’. A total of 215 miRNAs, containing 153 known miRNAs belonging to 57 families and 62 novel miRNA, were involved in flower colour formation. In particular, 55 miRNAs were significantly differently expressed. Based on miRNA–mRNA regulatory network, ath-miR5658 could affect the synthesis of pelargonidin, cyanidin and delphinidin through downregulating accumulation of anthocyanidin 3-O-glucosyltransferase; ath-miR868-3p could regulate isoflavonoid biosynthesis through downregulating expression of CYP81E1/E7; ath-miR156g regulated the expression of flavonoid 3',5'-hydroxylase; and ath-miR829-5p regulated flavonol synthasein flavonoid biosynthesis process. This research will provide important roles in breeding new varieties with rich flower colour.

## Teaser

Integration analysis of RNAomics and microRNAome of *Rhododendron simsii* Planchon flowers were performed to reveal the molecular mechanism of flower colour formation in three *R. simsii* varieties with red, pink and crimson flowers, respectively.DEGs were mainly involved in processes of ‘catalytic activity’, ‘binding’, ‘metabolic process’ and ‘cellular process’, as well as pathways of ‘metabolic pathways’, ‘biosynthesis of secondary metabolites’, ‘plant-pathogen interaction’ and ‘phenylpropanoid biosynthesis’. A total of 215 miRNAs were involved in flower colour formation and 55 were significantly differently expressed. Ath-miR5658 could affect the synthesis of pelargonidin, cyanidin and delphinidin through downregulating accumulation of anthocyanidin 3-O-glucosyltransferase, ath-miR868-3p could regulate isoflavonoid biosynthesis through downregulating expression of CYP81E1/E7, ath-miR156g regulated the expression of flavonoid 3',5'-hydroxylase and ath-miR829-5p regulated flavonol synthasein flavonoid biosynthesis process. This research will provide important roles in breeding new varieties with rich flower colour.

## Introduction

*Rhododendron*, a large vascular plant genus mainly distributed around the northern hemisphere, contains approximately 1000 species showing different ecological types, various flower colours and outstanding flower patterns (Bhattacharyya 2011; Popescu and Kopp 2013). *Rhododendron simsii* Planchon (2*n* = 26), a typical member of the *Rhododendron* genus, is critical for ecotourism and landscape greening due to massive and bright-colored flowers (Hahn *et al.* 2016). Widely used as outstanding ornamental and medicinal species in China, Western Europe, USA and Japan, *R. simsii* was introduced into Europe from Asian in the late 18th century (Wang *et al.* 2019). Rich flower colours have been observed in wild *R. simsii* germplasm resources due to the accumulation of flavonoids in petals, including white, red, carmine red, pink, purple and lilac, which shows great potential for developing new cultivars with desired characteristics. Therefore, *R. simsii* is often used for germplams innovation and breeding breakthrough varieties (Hahn *et al.* 2016; Wang *et al.* 2019).

Flower colour is controlled by integrated effects of pigment composition, cell pH, cell shape and co-pigmentation (Sinopoli *et al.* 2019; Li *et al.* 2023; Wang *et al.* 2023). Transcription factors, transcriptional co-regulators, proteins involved in epigenetic control of gene expression and microRNAs are all involved in flower colour formation (Guan *et al.* 2021; Ren *et al.* 2021; Selva *et al.* 2021; Takeda *et al.* 2023). In particular, microRNAs (miRNA) are a class of non-coding single-strand molecules negatively regulating gene expressions both at transcriptional and posttranscriptional levels via cleaving targets or repressing protein translation,which play important roles in flower colour formation (Allahverdy and Rashidi 2023; Takeda *et al.* 2023). Functional mature miRNAs are incorporated into Argonaute 1 (AGO1)-containing RNA-induced silencing complex, and cause mRNA degradation or translational repression in a sequence-specific manner (Abel *et al.* 2022). Therefore, the construction of miRNA–mRNA regulatory network might reveal molecular mechanisms underlying flower colour formation.

Particularly, research has long been focussed on the heredity of flower colours, doubleness of flowers and hose-in-hose character (coloured calyx) of *R. simsii* (Heursel 1981). Based on HPLC and LC–MS analyses, anthocyanins, quercetin 3-glucoside and quercetin 3-rhamnoside all contribute a lot to the reddish-purple blotches in upper inside of *R. simsii* red petals (Huyen *et al*. 2017). Flavones have also been isolated and well identified from *R. simsii* flowers, which could protect isolated rat heart from ischaemia-reperfusion injury and hippocampal neurone from hypoxia-reoxygenation injury (Sun *et al*. 2018; Guo *et al*. 2020). The 3-gene model could well describe most flower colour variation in azalea, but could not clarify pink-coloration (De Keyser *et al*. 2013). In total, 57 RsWRKYs randomly distributed on 13 chromosomes were identified in *R. simsii* genome data, which could regulate flowering in *R. simsii* (Wan *et al*. 2022).

The understanding of flower colour formation is vital for flower colour breeding of *R. simsii*. However, no systematic study of molecular mechanism underlying flowering colour formation has been conducted in *R. simsii*, which has greatly hampered the exploitation of important wild germplasm resources. In this study, mRNA and miRNA expression profiles of *R. simsii* flowers with different colours (red, pink and crimson) were systematically clarified, anthocyanin biosynthesis-related genes and regulatory miRNAs were screened, hoping to benefit the genetic improvement and molecular breeding of *R. simsii* and other *Rhododendron* species.

## Materials and Methods

### Plant materials

**Figure 1.**
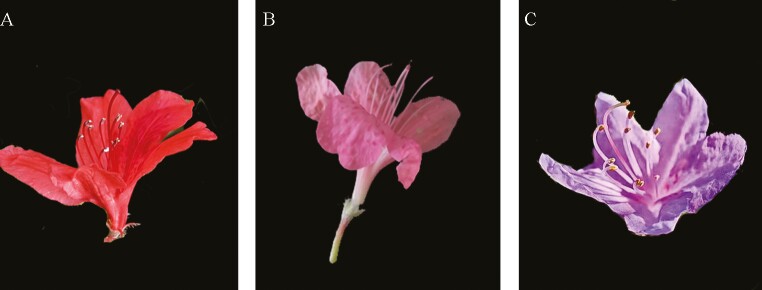
*Rhododendron simsii* flower tissues sampled for RNA-seq and miRNA-seq: (A) ‘Red variety’; (B) ‘Pink variety’ and (C) ‘Crimson variety’.

More than 50-year-old *R. simsii* plants were selected from the shrub ecosystem located in Dabie Mountains (30°58'15’’–31°02’’08’’N, 116°03’’15’’–116°09’’36’’E, 950–1020 m, April, 2023). Flower colours of *R. simsii* varieties were analysed with the help of RHS (Royal Horticultural Society) colour chart. Healthy red, pink and crimson flowers were separately sampled from *R. simsii* ‘Red variety’, ‘Pink variety’ and ‘Crimson variety’ during blooming period (Fig. 1). For each variety, flowers were collected from three individual plants and pooled together as one sample. Three biological repeats were prepared for each flower sample. Flower samples were immediately frozen in liquid nitrogen and stored at -80 °C until further extraction. All plant materials were conserved in Huanggang Normal University Herbarium (Huanggang, Hubei, China). All procedures were complied with the Convention on Biological Diversity.

### *Transcriptome sequencing, assembly, annotation and DEGs* id*entification*

Total RNA was isolated using TRIzol kit (Takara) following manufacturer’s instructions, treated with *DNase* I, enriched with magnetic oligo(dT) breads and fragmented by fragmentation buffer. First-stranded cDNA synthesis was conducted with fragmented mRNA and random hexamers. Following *RNase* H and DNA polymerase I-mediated second stand synthesis, double-stranded cDNAs was purified, end repaired and added with poly (A). After adaptor ligation, polymerase chain reaction (PCR) amplification (16 cycles) was carried out. The cDNA libraries were sequenced on MGI DNBSEQ-T7 platform. Raw reads were filtered with Fastp software (https://github.com/OpenGene/fastp). FastQC (http://www.bioinformatics.babraham.ac.uk/projects/fastqc) was used for quality control of sequence data before assembly. Moreover, Hisat2 was used to align clean reads to reference genome of *R. simsii* (ASM1428224v1, https://www.ncbi.nlm.nih.gov/datasets/genome/GCA_014282245.1/). The rMATS was used to detect differential alternative splicing events (Shen *et al.* 2014). Single nucleotide polymorphisms (SNPs) and InDels were identified with SAMtools and Bcftools. StringTie was used to assemble transcriptome (Pertea *et al.* 2015).

Trinity software was selected to extract open reading frames and predict potential protein-coding domain sequences. Functional annotation was obtained through the BLAST hit against Nr (NCBI non-redundant protein sequences), Nt (NCBI nucleotide sequences), GO (Gene ontology), KOG (euKaryotic Ortholog Groups), KEGG (Kyoto Encyclopedia of Genes and Genomes), Swiss-Prot and Pfam (Protein family) databases. Expression levels of assembled unigenes were calculated using FPKM (Fragments per kb per million mapped reads) method (Wang *et al.* 2018a). In particular, differentially expressed genes were screened out through R package DESeq2 (adjusted *P* value < 0.05 and FDR < 0.10) (Love *et al.* 2014).

### Small RNA library construction, sequencing and identification of miRNAs

To further clarify posttranscriptional gene regulation, small RNA library construction was carried out according to the protocol of NEBNext Multiplex Small RNA Library Prep Set, quantitative analysis was performed with Quant-iT PicoGreen dsDNA Assay Kit and sequencing was performed on MGI DNBSEQ-T7 platform (single-end). *Rhododendron simsii* genome (ASM1428224v1) served as reference genome. Mapped small RNA tags were analysed with Repeat Masker software to remove protein-coding genes, tRNA, rRNA, snRNA, snoRNA and tags derived from repeat sequences. Then, the remaining tags were aligned with known miRNAs in miRBase21.0 database, and modified with mirdeep2 software (Kozomara and Sam 2014). Softwares miREvo and mirdeep2 were used to predict novel miRNA through analysing secondary structure, minimum free energy and dicer cleavage site (Friedländer *et al.* 2012).

Target genes of miRNAs were predicted by psRNA Target server, and subjected to GO enrichment analysis implemented with GOseq and KEGG pathway analysis. Expression levels of miRNA were calculated with TPM (transcript per million) following formula ‘normalized expression = mapped read count/total reads×1 000 000)’. Differential expression analysis of miRNAs was performed with DESeq R package (1.8.3), in which *P* values was adjusted according to Benjamini and Hochberg method (Liu *et al.* 2019). Profiler package in the R platform was used to predict the potential functions of DEGs (Zou *et al.* 2019).

### Statistical analysis and real-time PCR amplification

TarBase (v.8; https://dianalab.e-ce.uth.gr/html/diana/web/index.php?r=tarbasev8) was applied to collect the empirically validated miRNA–mRNA pair data, which were integrated into a global miRNA–mRNA regulatory network. Nine miRNAs and potential target mRNAs were randomly selected for real-time qPCR amplification, including ath-miR159a, ath-miR166b-3p, ath-miR166c, ath-miR168a-3p, ath-miR319b, ath-miR396a-3p, ath-miR8175, novel_mir8 and novel_mir26. In particular, *actin* gene served as internal control. Gene-specific primers were designed with Primer 5 software [see Supporting Information—Table S1]. In particular, the selected differentially expressed miRNAs were validated with stem-loop qRT-PCR assay. Moreover, quantitative variation in different replicates was calculated with comparative threshold (CT) method (delta-delta CT, ΔΔCT) (Papadopoulos *et al.* 2016).

## Results

### Comparative transcriptome analysis among different colour varieties of *R. simsii*

#### Statistical analysis of RNA-seq data

Nine cDNA libraries with three repetitions for each variety were sequenced. Total reads were 42 265 228-78 751 278 for ‘Red variety’, 50 007 378-58 400 834 for ‘Pink variety’ and 62 882 854–74 057 210 for ‘Crimson variety’, respectively [see Supporting Information–Table S2]. Clean reads were obtained through removing adaptor, sequences with low quality and ambiguous sequences. The rates of clean reads were all higher than 99.902 %. In particular, more than 79.25 % clean reads could be mapped to the reference genome of *R. simsii* (ASM1428224v1), in which 3.56–3.86 % could be multiple mapped. Furthermore, the Q20 values were all more than 97.49 %, and GC contents ranged between 46.30 % and 46.81 % [see Supporting Information—Table S2]. Numbers of InDel and SNP markers were 16 196–20 856 and 403 918–508 043, respectively. ‘Exon skipping’ was the main alternative splicing events followed by ‘alternative 3' splice site’ and ‘alternative 5' splice site’, while ‘mutually exclusive exon’ was the least [see Supporting Information—Table S3]. In total, a set of 29 429 transcripts were obtained and assembled into 97 856 unigenes (72 632 054 bp) with an N50 value of 1223 bp. The average lengths of clean reads and unigenes were 785 bp and 726 bp, respectively. Expression levels of unigenes were mainly 0.1–3.75, as the ratio ranged between 29.55 % and 31.50 % [see Supporting Information—Table S4].

#### Analysis of differentially expressed mRNAs between different varieties

**Figure 2.**
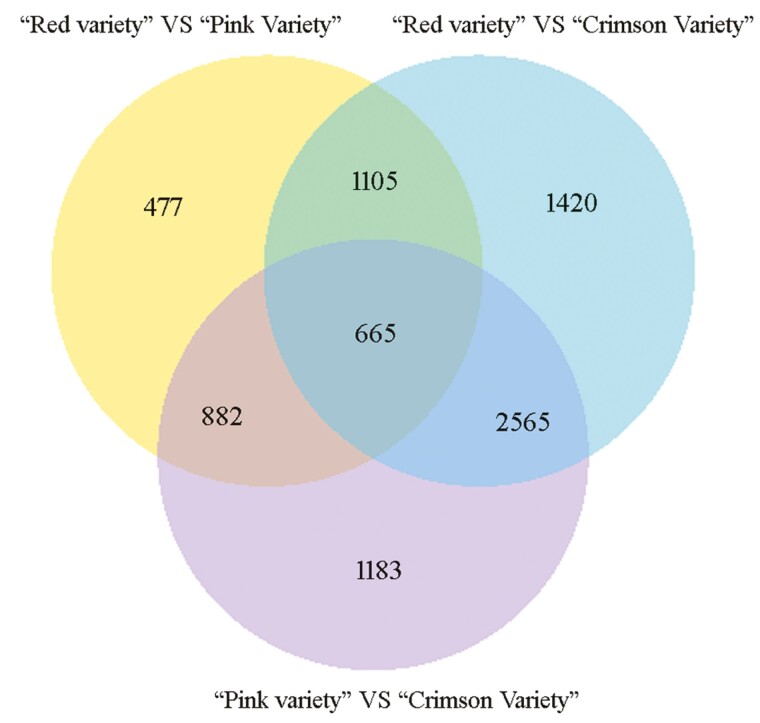
Venn diagram of differentially expressed unigenes among ‘Red variety’ versus ‘Pink variety’, ‘Red variety’ versus ‘Crimson variety’, and ‘Pink variety’ versus ‘Crimson variety’.

**Figure 3.**
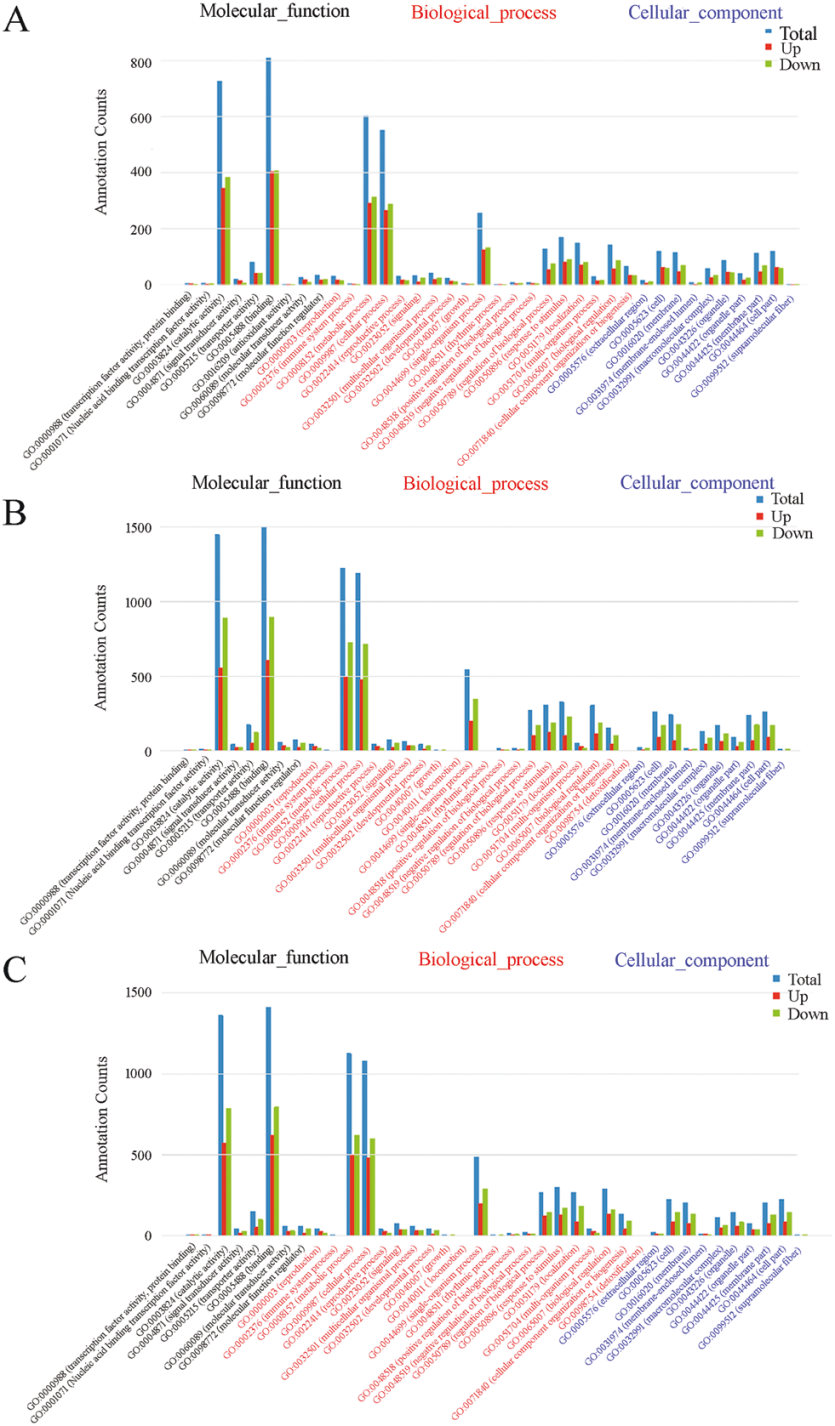
GO annotation analysis of target genes of differentially expressed miRNAs: (A) ‘Red variety’ versus ‘Pink variety’; (B) ‘Red variety’ versus ‘Crimson variety’; (C) ‘Pink variety’ versus ‘Crimson variety’. *X* axis indicated categories, *Y* axis represented counts of category.

**Figure 4.**
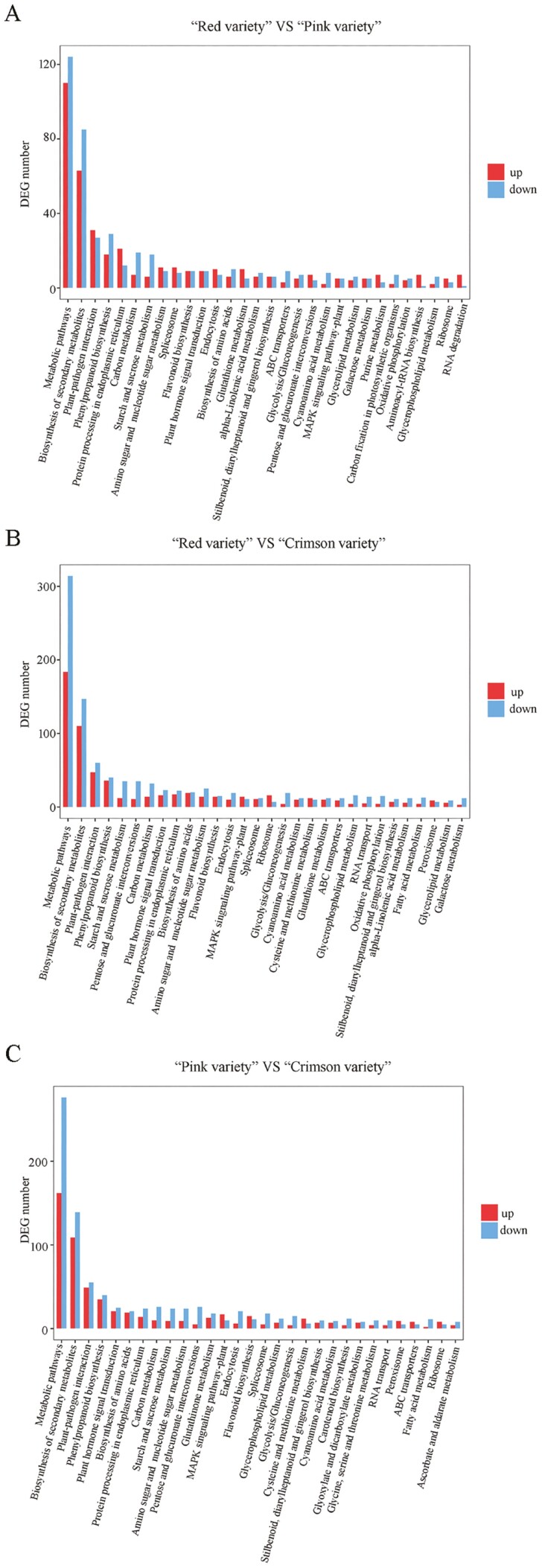
KEGG pathways analysis of target genes of differentially expressed miRNAs: (A) ‘Red variety’ versus ‘Pink variety’; (B) ‘Red variety’ versus ‘Crimson variety’; (C) ‘Pink variety’ versus ‘Crimson variety’. *X* axis refers to categories, *Y* axis represents counts of category.

**Figure 5.**
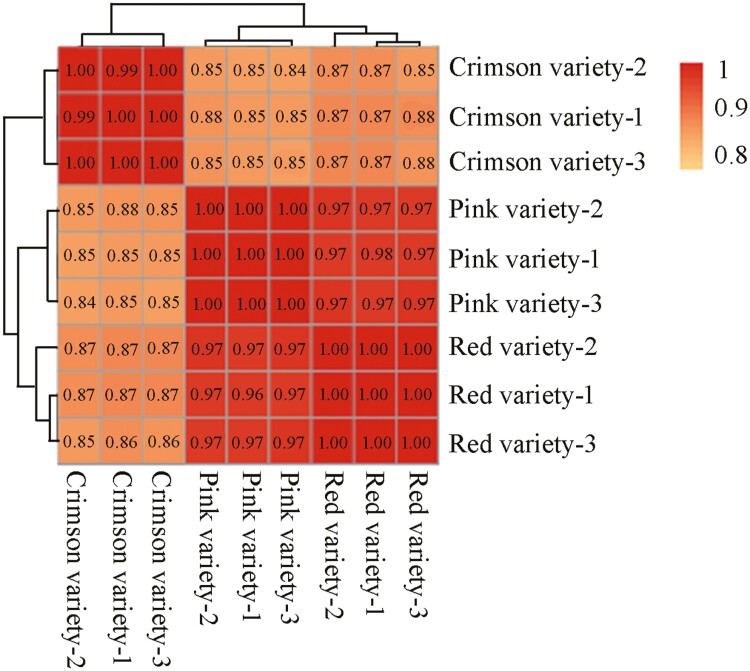
Correlation matrix analysis of ‘Red variety’, ‘Pink variety’ and ‘Crimson variety’ of *R. simsii* based on Pearson’s correlation coefficient (*P* value < 0.001, *FDR* < 0.001).

After data filtering (*P* value < 1E–3, FDR < 1E–3, and Fold Change > 2), 3129, 5755 and 5295 DEGs were identified between ‘Red variety’ and ‘Pink variety’, ‘Red variety’ and ‘Crimson variety’, as well as ‘Pink variety’ and ‘Crimson variety’, respectively (Fig. 2). Compared with ‘Red variety’, 1507 DEGs were up-regulated and 1622 DEGs were down-regulated in ‘Pink variety’ [see Supporting Information—Table S5A]. In comparison with ‘Red variety’, 2148 and 3607 DEGs were up-regulated and down-regulated in ‘Crimson variety’, respectively [see Supporting Information—Table S5B]. Compared with ‘Pink variety’, expression of 2089 GEGs was enhanced, while the expression of 3206 DEGs were down-regulated in ‘Crimson variety’ [see Supporting Information—Table S5C]. According to GO terms, these typical DEGs were involved in ‘catalytic activity’ (GO:0003824), ‘binding’ (GO:0005488), ‘metabolic process’ (GO:0008152) and ‘cellular process’ (GO:0009987) (Fig. 3). Furthermore, ‘metabolic pathways’ (map01100), ‘biosynthesis of secondary metabolites’ (map01110), ‘plant-pathogen interaction’ (map04626) and ‘phenylpropanoid biosynthesis’ (map00940) contributed a lot to the flower colour variation existing in *R. simsii* varieties (Fig. 4). Based on statistical analysis through calculating Pearson’s correlation coefficient (*r*) value, replicates in each sample showed a high correlation (Fig. 5). Compared with ‘Crimson variety’, ‘Red variety’ and ‘Pink variety’ had more association. Moreover, gene cluster inferred that DEGs could be clustered into two clades (Fig. 5). Good correlation (*R*^2^ = 0.8723) was observed between the RNA-seq data and qRT-PCR data.

## Small RNA meta-analysis among three varieties of *R. simsii*

### Statistical analysis of small RNA sequencing data

Removing low-quality reads and adaptor, 15 836 081-20 526 460, 793 802–17 324 699 and 11 947 123–15 428 901 clean reads (≧18nt) were obtained for ‘Red variety’, ‘Pink variety’ and ‘Crimson variety’, respectively [see Supporting Information—Table S6]. In particular, the Q20 and Q30 values were above 98.94 % and 96.82 %, respectively. GC contents varied from 55.34 % (‘Pink variety’-2) to 56.90 % (‘Crimson variety’-3). The percentage rate of mapped reads on *R. simsii* reference genome ranged from 67.68 % (‘Crimson variety’-1) to 89.89 % (‘Red variety’-3) [see Supporting Information—Table S6]. Moreover, similar length distributions existed in three *R. simisii* varieties [see Supporting Information—Fig. S1]. The majority of 18–36 nt small RNAs ranged from 21 to 24 nt in length. In particular, the most abundant sRNAs were 24 nt, followed by 21 nt and 22 nt. Particularly, rRNAs (853 418–1 354 362), snoRNA (25 279–35 637), snRNA (1494–3088), sRNA (17 499–26 859) and tRNA (3238–14 346) were successfully annotated through searching against GenBank, tRNAdb, Rfam, Repbase and Silva databases, respectively [see Supporting Information—Table S7]. Good correlation (*R*^2^ = 0.8687) was obtained between real-time PCR amplification and sequencing data, which further confirmed the high reliability of miRNA-seq in this research [see Supporting Information—Fig. S2].

### Id*entification of candidate miRNAs in R. simsii*

**Figure 6.**
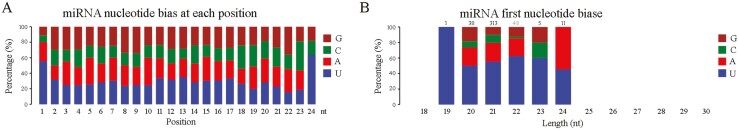
Nucleotide biases of known miRNAs in different positions (A) and first nucleotide biases of known miRNA in *R. simsii* flower (B).

Unannotated RNAs were subjected for miRNA identification. Totally, 153 known miRNAs belonging to 57 families were screened, and miR156 family was the largest represented family with 14 members, followed by miR166 (10 members), miR169 (8 members) and miR157 (7 members) [see Supporting Information—Table S8]. A total of 32 miRNA families had only one member. These known miRNAs sharing homology with Arabidopsis miRNAs were named according to that of *A. thaliana*. Bases at 1th and 24th had obvious uracil (U) base preference for known miRNA (Fig. 6). In particular, 102 miRNAs were all expressed in three verities [see Supporting Information—Table S8]. In addition, 10, 10 and 7 miRNAs were co-owned by ‘Red variety’ and ‘Pink variety’, ‘Red variety’ and ‘Crimson variety’, as well as ‘Pink variety’ and ‘Crimson variety’ [see Supporting Information—Table S8]. Furthermore, 5, 9 and 10 miRNAs were unique to ‘Red variety’, ‘Pink variety’ and ‘Crimson variety’, respectively [see Supporting Information—Table S9].

**Figure 7.**
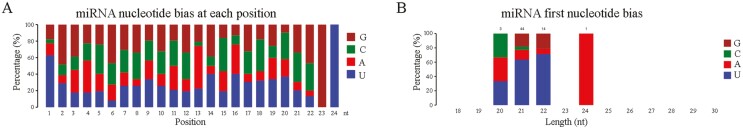
Nucleotide biases of novel miRNAs in different positions (A) and first nucleotide biases of novel miRNA in *R. simsii* flower (B).

These miRNA sequences, meeting threshold of miRDeep2 analysis without homologous miRNA listed in miRBase, were used to predict novel miRNAs (total score > 0). In total, 62 novel mature miRNA sequences were identified (Table S10). Particularly, four length types of miRNAs were predicted, including 21 nt (44, 70.97 %), 22 nt (14, 22.58 %), 20 nt (3, 4.84 %) and 24 nt (1, 1.61 %). For these novel miRNA, 1th and 24th bases had obvious U base preference, while G base preference existed at the 23th position (Fig. 7).

### Analysis of differentially expressed miRNAs between different varieties

**Figure 8.**
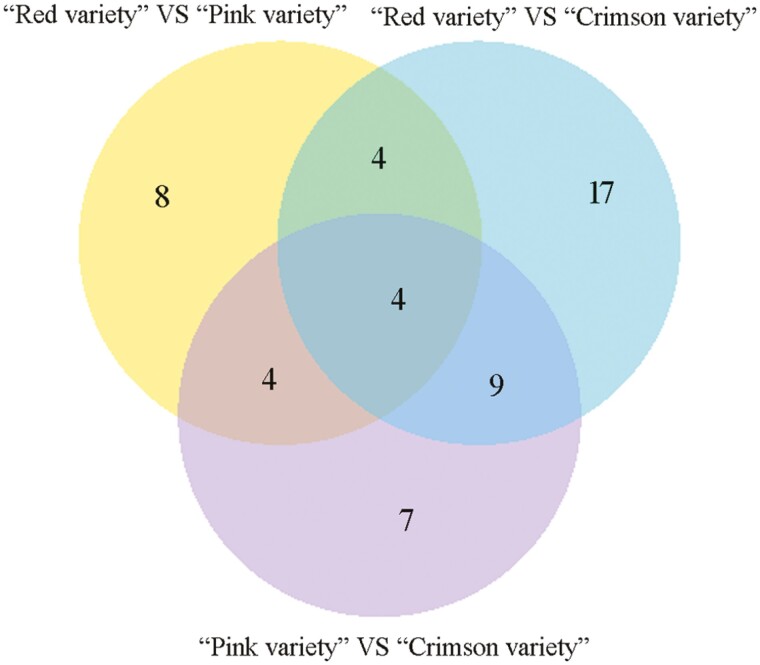
Venn diagram of differentially expressed miRNAs among ‘Red variety’ versus ‘Pink variety’, ‘Red variety’ versus ‘Crimson variety’ and ‘Pink variety’ versus ‘Crimson variety’.

**Figure 9.**
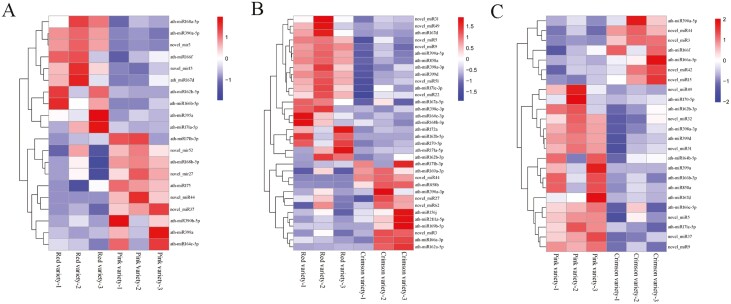
Cluster analyses of differently expressed miRNAs between different varieties: (A) ‘Red variety’ and ‘Pink variety’; (B) ‘Red variety’ and ‘Crimson variety’; (C) ‘Pink variety’ and ‘Crimson variety’.

Based on fold change (≥1 or ≤-1) and *P* value (<0.05) criteria, 53 miRNAs were significantly differently expressed among three varieties (Fig. 8). Compared with ‘Red variety’, l0 miRNAs were up-regulated and 10 miRNAs were down-regulated in ‘Pink variety’ see Supporting Information—Table S11A]; while the downregulated and upregulated miRNAs were 21 and 13 in ‘Crimson variety’, respectively see Supporting Information—Table S11B]. In relation to ‘Pink variety’, the expression levels of 17 miRNAs were downregulated, while expression levels of 7 miRNAs were upregulated in ‘Crimson variety’ [see Supporting Information—Table S11C]. Based on miRNA cluster analysis, novel_miR15, ath-miR167d, ath-miR162b-5p and ath-miR166b-5p were highly expressed in ‘Red variety’ (Fig. 9A); ath-miR390a-3p, ath-miR156j, ath-miR2111a-5p and ath-miR169b-5p had higher expression levels in ‘Crimson variety’ (Fig. 9B); ath-miR399a, ath-miR167d, novel_miR49 and ath-miR170-5p were highly expressed in ‘Pink variety’ (Fig. 9C).

### Target gene prediction and enrichment analysis

Totally, 2889 miRNA-target site (386 target genes) were obtained by predicting with transcriptome data as the target database. Among the miRNA–target mRNA pairs, 79.97 % miRNA could target multiple unigenes and the numbers ranged from 1 to 40 [see Supporting Information—Table S12]. Particularly, negative correlation was observed between the expression levels of miRNAs and corresponding target genes. These miRNA–mRNA pairs might partially regulate flower colour formation in *R. simsii*, as flower colour was comprehensively modulated by multiple factors.

Compared among three different varieties, genes involved in biologic processes of ‘meristem maintenance’ (GO:0010073), ‘meristem development’ (GO:0048507), ‘protein glycosylation’ (GO:0006486), ‘protein ubiquitination’ (GO:0016567), ‘carbohydrate metabolic process’ (GO:0005975), ‘glutathione metabolic process’ (GO:0006749), ‘DNA repair’ (G0:0006281), ‘signal transduction’ (GO:0007165) and ‘translation’ (GO:0006412) contributed a lot to flower colour variation [see Supporting Information—Table S13]. Target genes associated with cellular component of ‘nucleus’ (GO:0005634), ‘extracellular space’ (GO:0005615) and ‘cCAAT-binding factor complex’ (GO:0016602) corresponded for flower colour variation of red, pink and crimson [see Supporting Information—Table S13]. Furthermore, genes related to molecular function of ‘lipid binding’ (GO:0008289), ‘GTP binding’ (GO:0005525), ‘hemebinding’ (GO:0020037), ‘glutathione transferase activity’ (GO:0004364), ‘hydrolase activity, hydrolysing O-glycosyl compounds’ (GO:0004553), ‘ATP hydrolysis activity’ (GO:0016887) and ‘glycosyltransferase activity’ (GO:0016757) might also play important roles in flower colour formation in *R. simsii* [see Supporting Information—Table S13]. Through KEGG pathway enrichment analysis, genes involved in pathways of ‘monobactam biosynthesis’ (map00261), ‘selenocompound metabolism’ (map00450), ‘sulfur metabolism’ (map00920), ‘purine metabolism’ (map00230), ‘plant-pathogen interaction’ (map04626), ‘MAPK signalling pathway-plant’ (map04016), ‘glutathione metabolism’ (map00480), ‘phenylpropanoid biosynthesis’ (map00940) and ‘starch and sucrose metabolism’ (map00500) might regulate flower colour formation.

## Integrative analysis of RNA-seq and miRNA-seq

### *MiRNA*–*mRNA interaction network analysis*

Expression of genes mainly involved in biologic process of ‘meristem maintenance’ (GO:0010073), ‘meristem development’ (GO:0048507), ‘protein glycosylation’ (GO:0006486), ‘protein ubiquitination’ (GO:0016567), ‘response to oxidative stress’ (GO:0006979) and ‘hydrogen peroxide catabolic process’ (GO:0042744), involved in cellular component of ‘nucleus’ (GO:0005634) and ‘extracellular space’ (GO:0005615), involved in molecular function of ‘lipid binding’ (GO:0008289), ‘GTP binding’ (GO:0005525), ‘peroxidase activity’ (GO:0004601), ‘transferase activity’ (GO:0016758) and ‘serine-type endopeptidase inhibitor activity’ (GO:0004867) contributed to the red and pink variation in flower colour [see Supporting Information—Table S13A and see Supporting Information—Fig. S3). Particularly, these miRNA–mRNA regulated 16 pathways, especially ‘monobactam biosynthesis’ (map00261), ‘selenocompound metabolism’ (map00450), ‘sulfur metabolism’ (map00920), ‘purine metabolism’ (map00230), ‘plant-pathogen interaction’ (map04626) and ‘MAPK signalling pathway-plant’ (map04016).

Regarding to red and crimson variation of flower colour, genes were mainly involved in biologic process of ‘carbohydrate metabolic process’ (GO:0005975), ‘glutathione metabolic process’ (GO:0006749), ‘DNA repair’ (G0:0006281), ‘hydrogen peroxide catabolic process’ (GO:0042744) and ‘response to oxidative stress’ (GO:0006979); regulating cellular component of ‘nucleus’ (GO:0005634), ‘cCAAT-binding factor complex’(GO:0016602) and ‘extracellular space’ (GO:0005615); as well as related to molecular function of ‘hemebinding’ (GO:0020037), ‘glutathione transferase activity’ (GO:0004364), ‘hydrolase activity, hydrolysing O-glycosyl compounds’ (GO:0004553), ‘hexosyltransferase activity’ (GO:0016758), ‘UDP-glycosyltransferase activity’ (GO:0008194), ‘serine-type endopeptidase inhibitor activity’ (GO:0004867) and ‘peroxidase activity’ (GO:0004601) [see Supporting Information—Fig. S4 and see Supporting Information—Table S13B]. Furthermore, the miRNA–mRNA mainly regulated metabolic pathways of ‘plant-pathogen interaction’ (map04626), ‘MAPK signalling pathway-plant’ (map04016), ‘glutathione metabolism’ (map00480) and ‘phenylpropanoid biosynthesis’ (map00940).

A list of genes, involved in biological process of ‘signal transduction’ (GO:0007165), ‘protein glycosylation’ (GO:0006486), ‘carbohydrate metabolic process’ (GO:0005975) and ‘translation’ (GO:0006412), regarding to the cellular component of ‘nucleus’ (GO:0005634), ‘extracellular space’ (GO:0005615) and ‘nucleus’ (GO:0005634), as well as regulating molecular function of ‘ATP hydrolysis activity’ (GO:0016887), ‘lipid binding’ (GO:0008289), ‘glycosyltransferase activity’ (GO:0016757) and ‘serine-type endopeptidase inhibitor activity’ (GO:0004867), contributed a lot the pink and crimson variation of *R. simsii* flower [see Supporting Information—Table S13C and see Supporting Information—Fig. S5]. These miRNA–mRNA significantly regulated pathways of ‘plant-pathogen interaction’ (map04626), ‘MAPK signalling pathway-plant’ (map04016) and ‘starch and sucrose metabolism’ (map00500).

### *Analysis of miRNA*–*mRNA associated with anthocyanin synthesis*

In *R. simsii* flowers, pelargonidin, cyanidin and delphinidin were major anthocyanins, which might be regulated by anthocyanin biosynthetic genes, including 5 homologous of phenylalanine ammonialyase (*PAL*) genes, 13 homologous of chalcone synthase (*CHS*) gene, 3 homologous of flavonoid 3-hydroxylase (*F3H*) genes, 5 homologous of flavonoid 3',5'-hydroxylase (*F3*'*,5*'*H*) genes, 2 homologous of flavonoid 3'-monooxygenase, 3 homologous of flavonoid O-methyltransferase, 1 homologous of anthocyanidin synthase (*ANS*) genes, 1 homologous of anthocyanidin reductase (*ANR*), 3 homologous of flavonoid O-methyltransferase (*CROMT2*), 6 homologous of flavonol synthase and 4 homologous of *DFR* genes [see Supporting Information—Table S7]. Furthermore, *PAL* and *CHS* genes also played great roles in ‘L-phenylalanine catabolic process’ (GO:0006559) and biosynthetic process (GO:0009058), respectively.

For *R. simsii*, 2 transcripts of phytochrome A (*PHYA*) genes, 1 transcripts of phytochrome B (*PHYB*) gene, 2 homologous of *FLOWERING LOCUS T* (*FT*) genes were involved in the photoperiod pathway. *FT* genes might contribute to regulation of ‘flower development’ (GO:0009909), ‘photoperiodism and flowering’ (GO:0048573) and ‘phosphatidylethanolamine binding’ (GO:0008429). In particular, *PHYB* and *PHYA* might be involved in the process of ‘signal transduction’ (GO:0007165), ‘regulation of transcription’ (GO:0006355), ‘detection of visible light’ (GO:0009584), ‘red, far-red light phototransduction’ (GO:0009585), ‘protein-tetrapyrrole linkage’ (GO:0017006) and ‘transcription coregulator activity’ (GO:0003712). In addition, three homologous of *DELLA* genes, associated with ‘transcription coregulator activity’ (GO:0003712), might regulated GA pathway.

In total, 138 homologous of *MYB* genes, 2 homologous of WD40 protein genes, 7 homologous of *WRKY* genes (*WRKY1*, *WRKY2*, *WRKY22* and *WRKY23*), 8 homologous of transcription factor *MYC2* and 24 homologous of *MADS-box* transcription factor were screened to regulate flower colour formation in *R. simsii* [see Supporting Information—Table S14]. Besides regulation of transcription, these *MYB* genes could also regulate ‘nucleosome assembly’ (GO:0006334). It is worth noting that *MADS-box* transcription factor could positively regulate processes of ‘RNA polymerase II’ (GO:0045944), ‘regulation of transcription’ (GO:0006355), ‘DNA binding’ (GO:0003677), ‘protein dimerization activity’ (GO:0046983), ‘RNA polymerase II transcription regulatory region sequence-specific DNA binding’ (GO:0000977) and ‘DNA-binding transcription factor activity’ (GO:0003700). These transcription factors might regulate the synthesis of coloured anthocyanins through multiple signal pathways.

**Figure 10.**
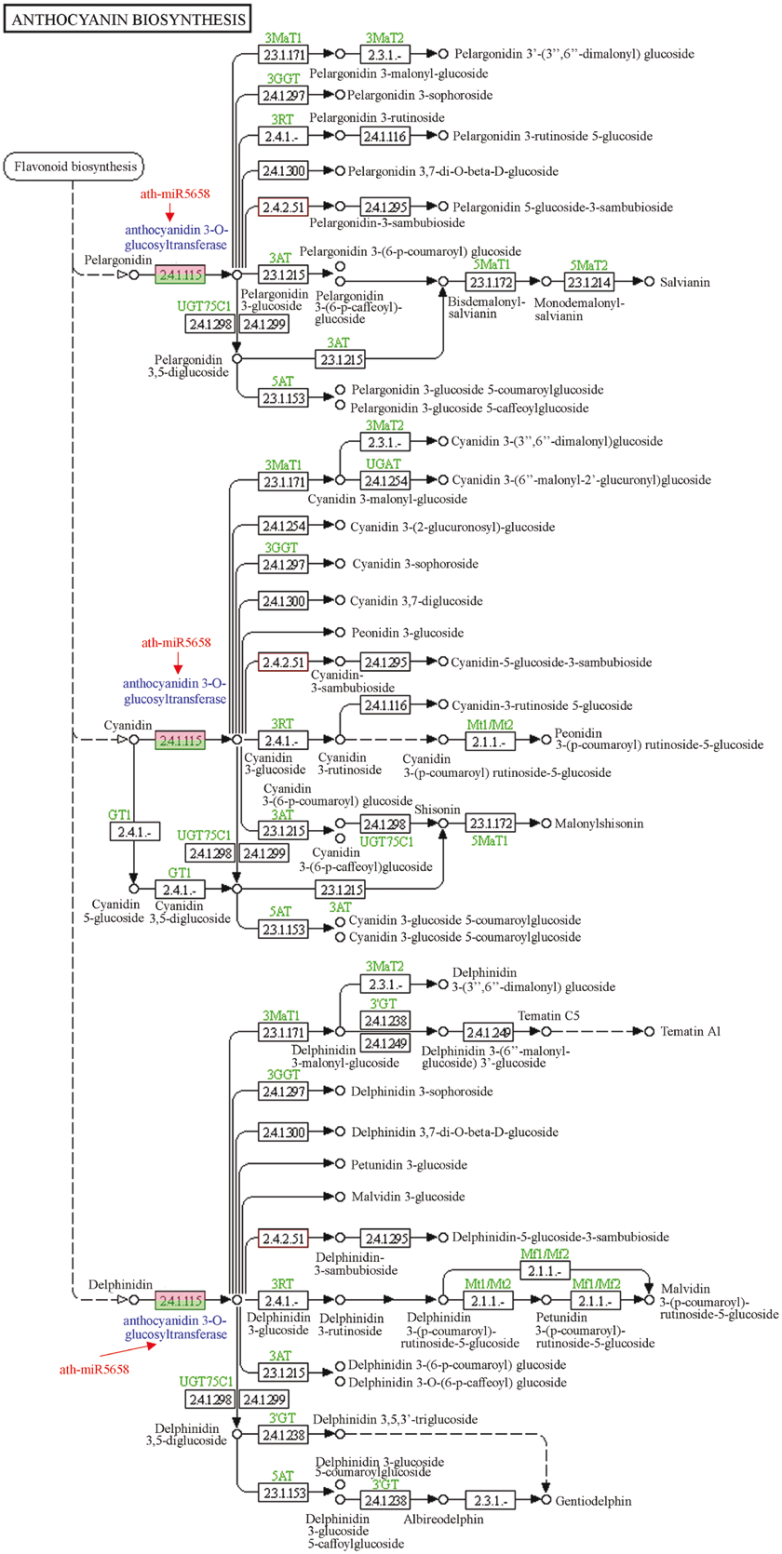
Overview of anthocyanin synthesis process in *R. simsii* flowers. Notes: numbers in the box referred to enzymes listed in Enzyme Commission hierarchy.

**Figure 11.**
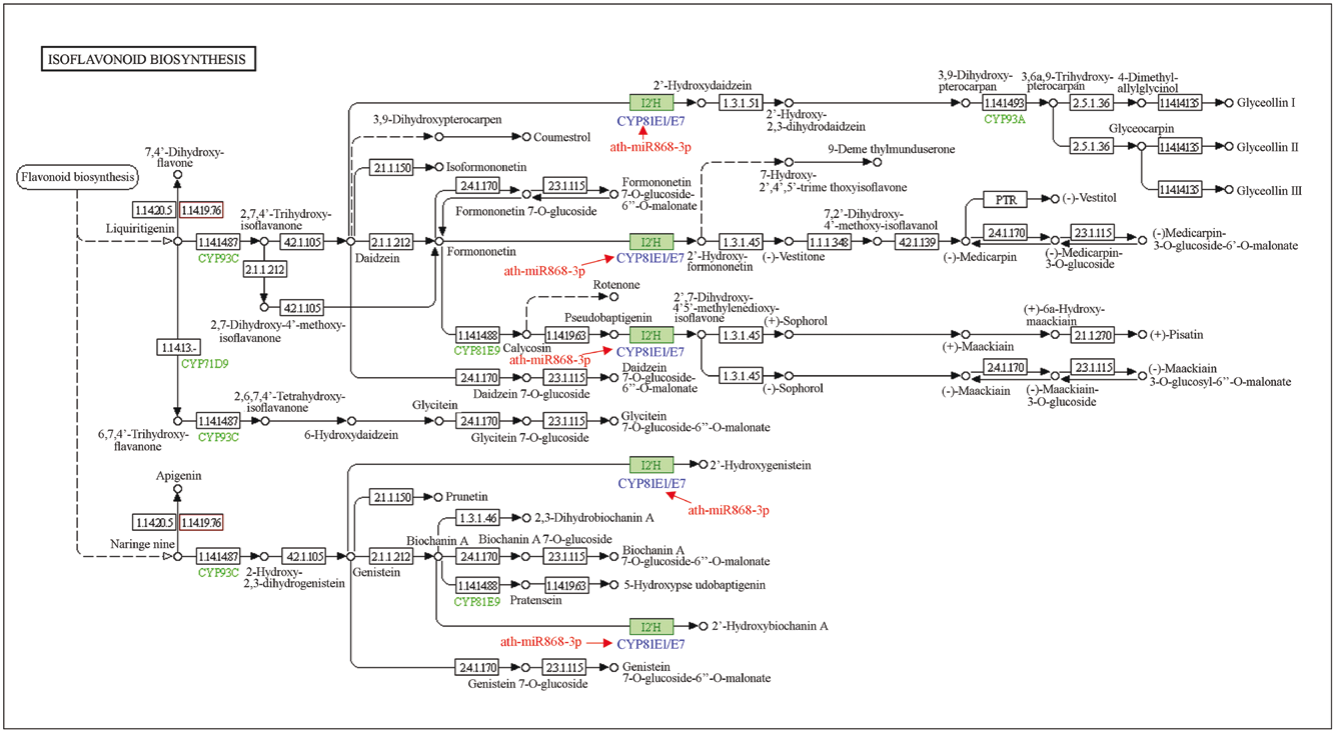
Overview of isoflavonoid biosynthesis process in *R. simsii* flowers. Notes: numbers in the box referred to enzymes listed in Enzyme Commission hierarchy.

**Figure 12.**
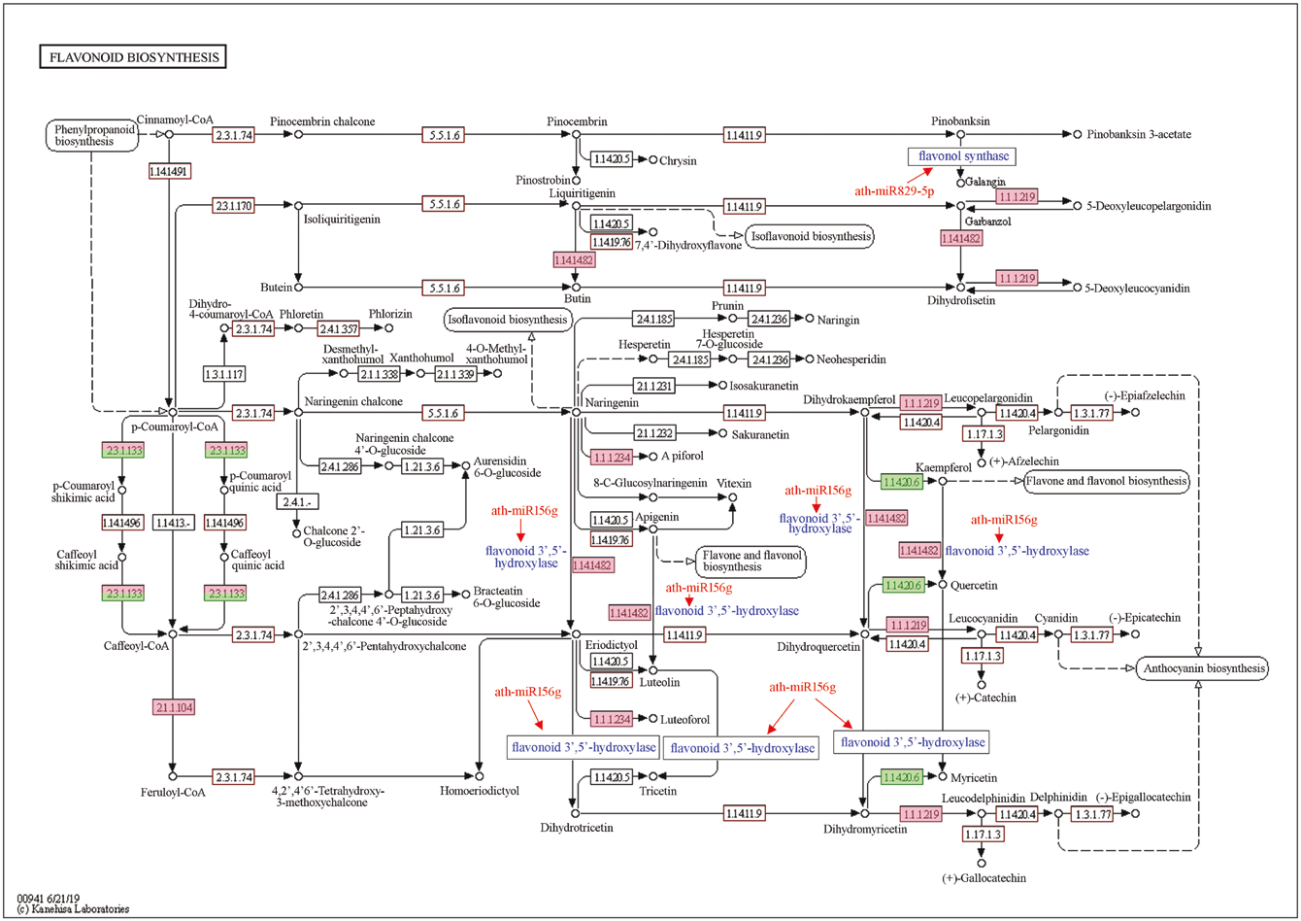
Overview of flavonoid biosynthesis process in *R. simsii* flowers. Notes: numbers in the box referred to enzymes listed in Enzyme Commission hierarchy.

Based on the integrative analysis of RNA-seq data and miRNA-seq data, ath-miR5658 could affect the synthesis of pelargonidin, cyanidin and delphinidin through downregulating the accumulation of anthocyanidin 3-O-glucosyltransferase in the anthocyanin synthesis process (Fig. 10). Particularly, ath-miR868-3p could regulate isoflavonoid biosynthesis through downregulating the expression of *CYP81E1/E7* gene, which encoded isoflavone/4'-methoxyisoflavone 2'-hydroxylase. During the process of isoflavonoid biosynthesis, CYP81E1/E7 are vital in catalysing the synthesis of 2'-hydroxyformononetin, 2'-hydroxydaidzein and 2',7-dihydroxy-4',5'-methylenedioxy-isoflavone from daidzein; while converting genistein into 2'-hydroxygenistein and 2'-hyroxybiochanin A, respectively (Fig. 11). For the flavonoid biosynthesis, naringenin could be converted into eriodictyol by flavonoid 3',5'-hydroxylase. Then, flavonoid 3',5'-hydroxylase converted eriodictyol into dihydrotricetin. In particular, the expression of flavonoid 3',5'-hydroxylase could be regulated by ath-miR156g (Fig. 12). Furthermore, the conversion of pinobanksin to galangin was catalysed by flavonol synthase, which could be regulated by ath-miR829-5p in the flavonoid biosynthesis process. Furthermore, ath-miR5658 was only expressed in ‘Pink variety’ and ‘Crimson variety’, ath-miR156g was present in ‘Red variety’ and ‘Crimson variety’, ath-miR829-5p existed only in ‘Pink variety’ and ‘Crimson variety’, while ath-miR868-3p was just expressed in ‘Red variety’ and ‘Pink variety’. In relation to carotenoid, flavone and flavonol biosynthesis, no typical miRNA was found to regulate corresponding mRNA expression.

## Discussion

Anthocyanins, one type of important secondary metabolites, are responsible for orange, red, magenta, crimson and blue petal or pulp colours of plant, which are regulated by multiple enzymes and transcription factors (Li *et al.* 2018; Gao *et al*. 2021; Yang *et al.* 2021). Particularly, *PAL*, *CHS*, *ANS*, *DFR* and *ANR* genes are well described to be involved in anthocyanin biosynthesis (Jiang *et al.* 2018). In *Acer pseudosieboldianum*, a colored-leaf tree native to Northeastern China, cyanidin 3, 5-O-diglucoside played important roles for final leaf colour, which was regulated by structural genes (*PAL*, *ANS*, *DFR*, *F3*'*H*) and regulatory genes (*MYB*, *bHLH* and *WD40*) (Gao *et al*. 2021). In *R. simsii* flowers, expression of these structural enzymes differed largely in three varieties. In particular, expression levels of 5 homologous of *F3*'*5*'*H* genes were higher in ‘Crimson variety’ than that of ‘Red variety’ and ‘Pink variety’.

Expression products of *F3*'*H* gene could significantly accelerate the accumulation of red cyanidin in purple and red root radishes (Masukawa *et al.* 2019). However, no homologous of *F3*'*H* gene could be detected in *R. simsii* flowers. DFR is a key enzyme catalysing the conversion of dihydroflavonol to colourless geranium, delphinium and cyanidin (Gao *et al.* 2021). In three *R. simsii* varieties, the homologous of *DFR* genes were differentially expressed. *ANS* genes are vital for the conversion of colourless proanthocyanidins into coloured anthocyanidins (Springob *et al.* 2003). Likewise, the homologous of *ANS* genes may play important roles in the synthesis of coloured anthocyanidins in *R. simsii* flowers. MYB proteins, widely involved in regulating plant developmental processes, could significantly upregulate the expression level of anthocyanin biosynthesis genes (Xu *et al.* 2015; Jian *et al.* 2019). Particularly, 138 homologous of *MYB* genes were found, which could enhance anthocyanin accumulation in *R. simsii* flowers.

Plant genome possess conserved small non-coding microRNAs genes, which could regulate various developmental programs of plants (Arazi and Khedia 2022; Guo *et al.* 2022; Teotia *et al.* 2023). In total, 153 known miRNAs (57 families) and 62 novel mature miRNA sequences were successfully screened, which might exert comprehensive regulation of flower colour formation in *R. simsii*. Most miRNAs identified in *R. simsii* flowers ranged from 20 nt to 24 nt, in accord with typical characteristics of dicer enzymatic cleavage. In particular, miR156 family, miR166 family, miR169 family and miR157 family were the main types, which have been reported to regulate many aspects of plant development and stress responses (Ritika *et al.* 2021). The miR156 can regulate expression of transcription factors SPL10 and SPL11, which could affect the early morphogenesis of *Arabidopsis* embryos (Nodine and Bartel 2010). The 24 nt miRNAs tended to start with 5'-U, while the 22 nt miRNAs preferred to have U base at 5' end. Likewise, the 24nt miRNAs in *Camellia oleifera* and *C. meiocarpa* tended to start with 5'-A, while 21nt miRNAs preferred to start with 5'-U (Feng *et al.* 2017). Differences existed between miRNA profiles of three *R. simsii* varieties may partially contribute to the variation in flower colour.

As a unique class of short endogenous RNAs, miRNAs may regulate target gene expression at post-transcriptional level through sequence complementary (Islam *et al.* 2022; Pei *et al.* 2023). In *R. simsii* flowers, ath-miR5658 could affect the synthesis of pelargonidin, cyanidin and delphinidin via downregulating accumulation of anthocyanidin 3-O-glucosyltransferase. In addition, miR5658 could regulate the posttranscriptional salt-stress responses, and showed great potentials for the molecular-assisted resistance breeding in *Tamarix* plants (Wang *et al.* 2018b). The ath-miR868-3p could regulate isoflavonoid biosynthesis through downregulating the expression of *CYP81E1/E7* gene, while ath-miR829-5p could regulate the expression of flavonol synthase gene, which affected the conversion of pinobanksin to galangin in *R. simsii* flower. Meanwhile, miRNA-829 regulated the expression of transcription factors, cell division regulating proteins and virus response gene in cotton (Khan Barozai *et al.* 2008).

*Rhododendron simsii*, an outstanding ornamental and medicinal species, shows great potential as breeding parents of commercial varieties. According to metabolome data of *R. simsii* flower, 79 flavonoids were obtained, including anthocyanidins (7), ﬂavanones (42), flavans (10), flavones (13) and flavonols (7) (Li *et al.* 2024). Moreover, isoflavonoids, 2-arylbenzofuran flavonoids, kuromanin, procyanidin (A2, B1 and B2), tulipanin also contributed to flower colour formation in *R. simsii* (Li *et al.* 2024). The miRNA-mediated regulatory networks and molecular mechanism in flower colour formation of *R. simsii* will accelerate the progress of molecular breeding. The datasets and results will provide important scientific resources and a basis for the genetic improvement of *R. simsii* varieties with novelty colours.

## Supporting Information

The following additional information is available in the online version of this article –

**Table S1.** Primer sequences of miRNAs and target genes used in real-time PCR analysis.

**Table S2.** Statistic analysis of RNA-seq in three *R. simsii* varieties with different flower colours.

**Table S3.** Alternative splicing events existed between different varieties.

**Table S4.** Expression level of unigenes in three different *R. simsii* varieties.

**Table S5.** Differently expressed genes among three different *R. simsii* varieties.

**Table S6.** Statistic analysis of siRNA-seq in three different *R. simsii* varieties.

**Table S7.** Distribution of small RNA sequencing among three varieties of *R. simsii*.

**Table S8.** Expression pattern of known miRNA in three different varieties of *R. simsii*.

**Table S9.** Expression pattern of miRNA in three different varieties of *R. simsii*.

**Table S10.** Information on novel miRNAs of three *R. simsii* varieties.

**Table S11.** Differentially expressed miRNAs among three *R. simsii* varieties.

**Table S12.** Differently expressed miRNA among three different *R. simsii* varieties.

**Table S13.** GO enrichment analysis of target genes of different expressed miRNAs among different *R. simsii* varieties.

**Table S14.** Information on structural genes and transcription factors associated with anthocyanin synthesis.

**Figure S1.** Length distributions of miRNAs in three *R. simsii varieties.*

**Figure S2.** GO enrichment of DEGs between ‘Red variety’ and ‘Pink variety’: (A) biological process and (B) molecular function.

**Figure S3.** GO enrichment of DEGs between ‘Red variety’ and ‘Crimson variety’: (A) biological process, (B) cellular component and (C) molecular function.

**Figure S4.** GO enrichment of DEGs between ‘Pink variety’ and ‘Crimson variety’: (A) cellular component and (B) molecular function.

**Figure S5.** Expression analysis of eight selected miRNAs and corresponding target genes by real-time PCR.

plae053_suppl_Supplementary_Figures

plae053_suppl_Supplementary_Tables

## Data Availability

BioProject, Biosample and SRA numbers for *R. simisii* mRNAs were PRJNA998472, SAMN36704916-SAMN36704933 and SRR25433158-SRR25433172, respectively. In addition, the associated BioProject, Biosample and SRA numbers for miRNAs were PRJNA995424, SAMN36470457-SAMN36470474 and SRR25301655-SRR25301672, respectively.
