# Supplementary material for: Molecular mechanism of flower colour formation in Rhododendron simsii Planchon revealed by integration of microRNAome and RNAomics
Source: AoB Plants. 2024 Oct 14;16(5):plae053. doi: 10.1093/aobpla/plae053 (PMC11489732; doi:10.1093/aobpla/plae053)
Supplement: plae053_suppl_Supplementary_Figures [file plae053_suppl_supplementary_figures.pdf]

Supplemental Figures and Tables

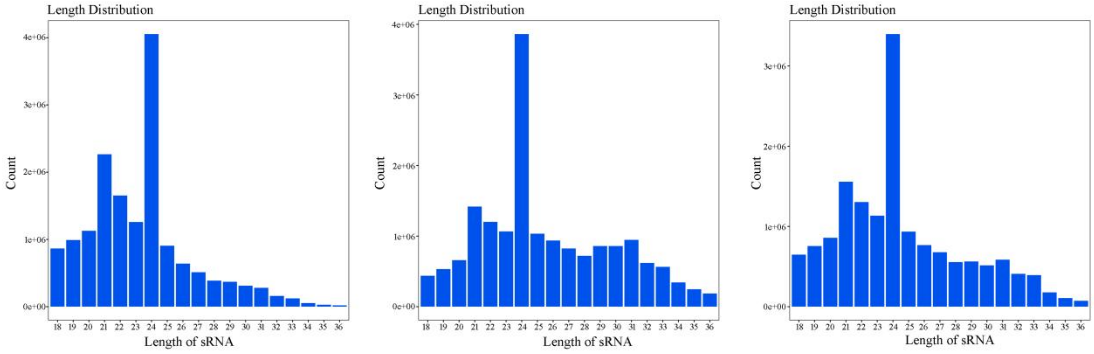

Figure S1 Length distributions of miRNAs in three *R. simsii varieties.*

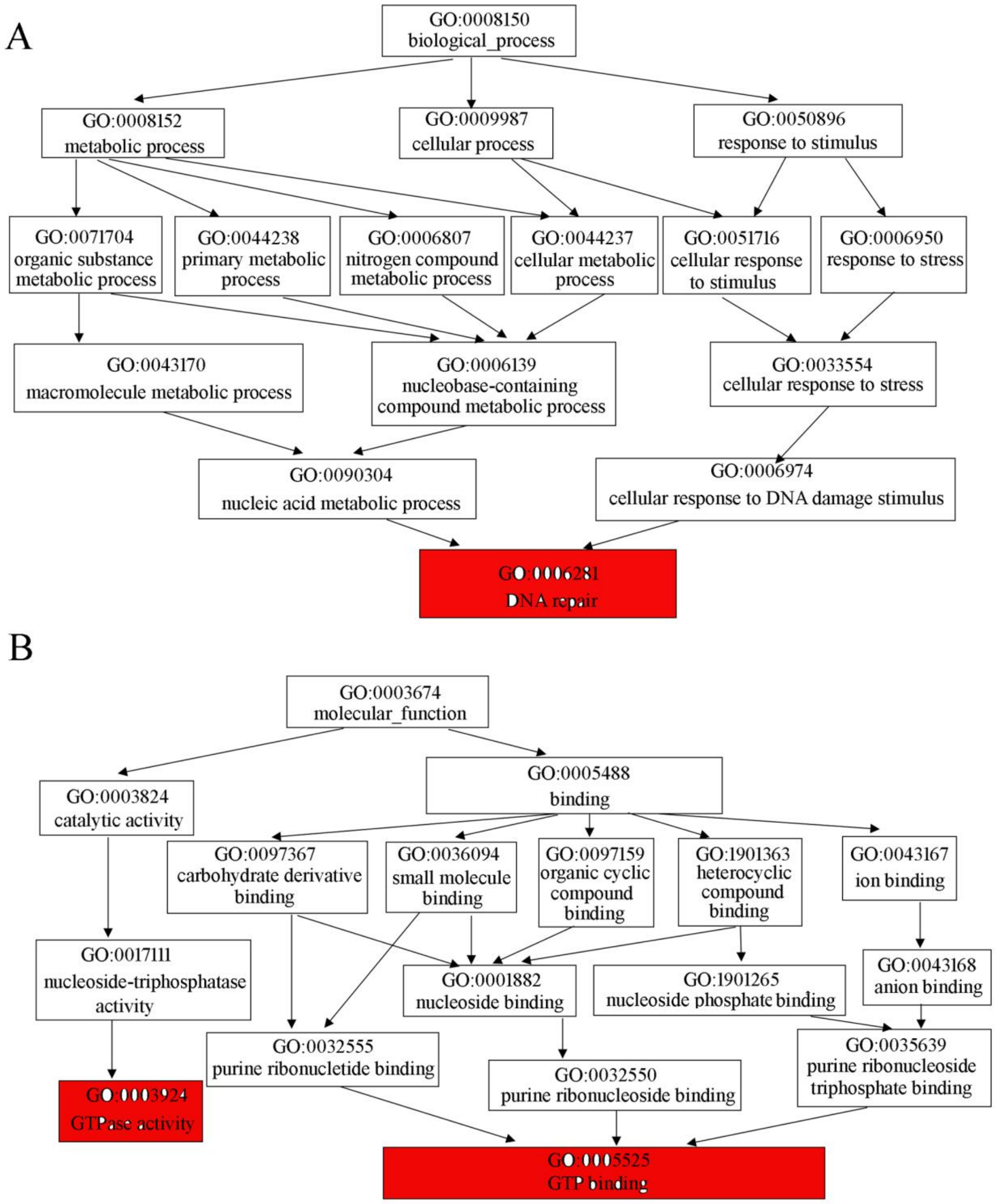

Figure S2 GO enrichment of DEGs between ‘Red variety’ and ‘Pink variety’: (A) biological process and (B) molecular function.

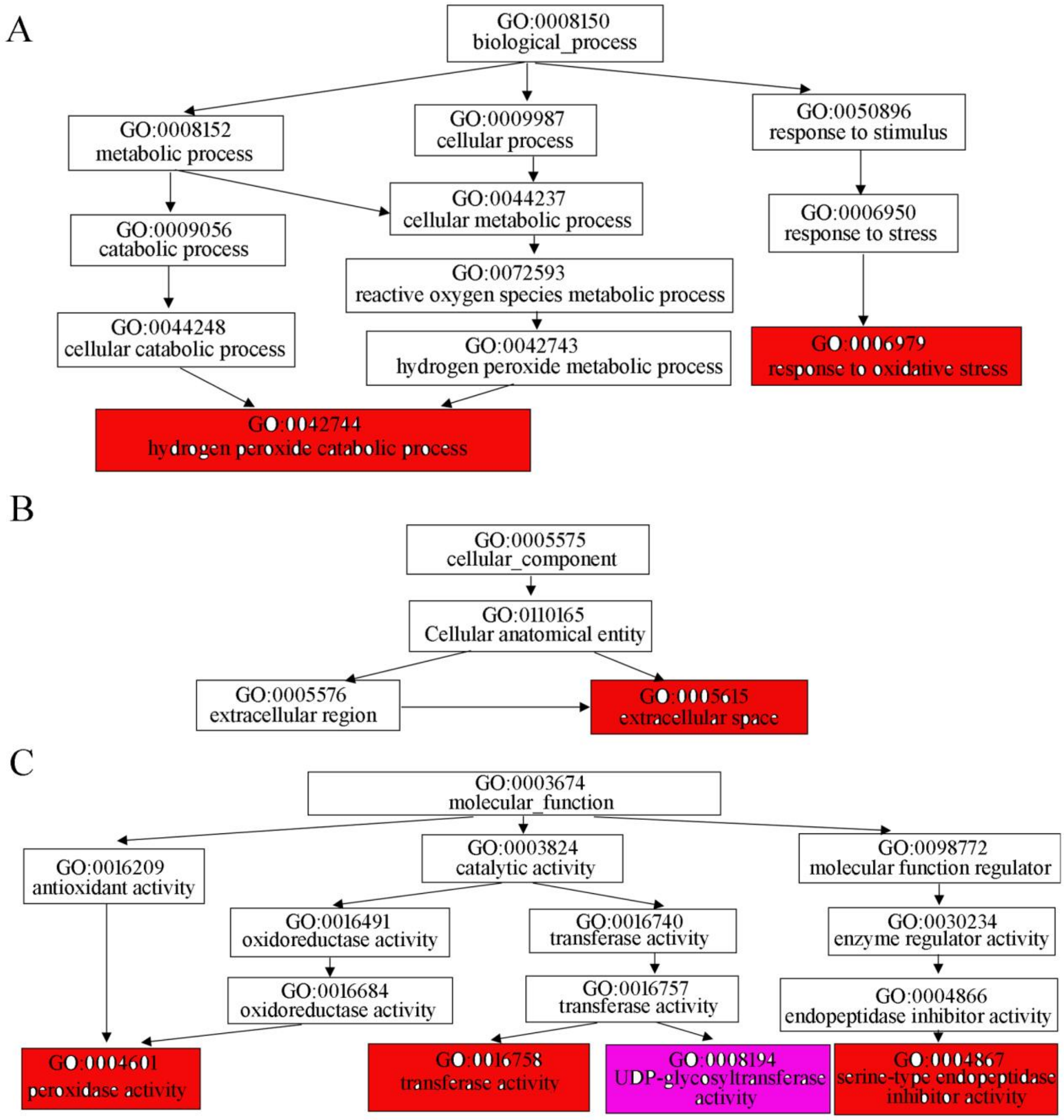

Figure S3 GO enrichment of DEGs between 'Red variety' and 'Crimson variety': (A) biological process, (B) cellular component, and (C) molecular function.

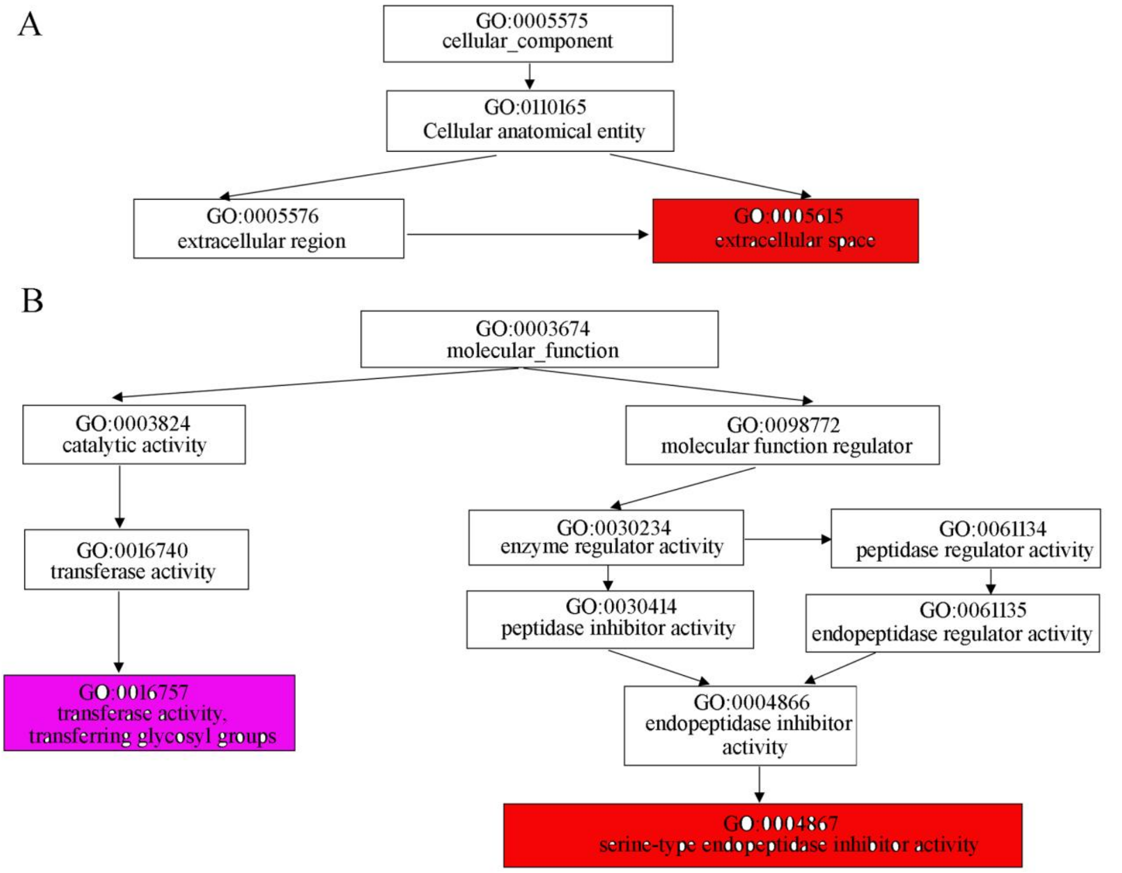

Figure S4 GO enrichment of DEGs between 'Pink variety' and 'Crimson variety': (A) cellular component and (B) molecular fucntion.

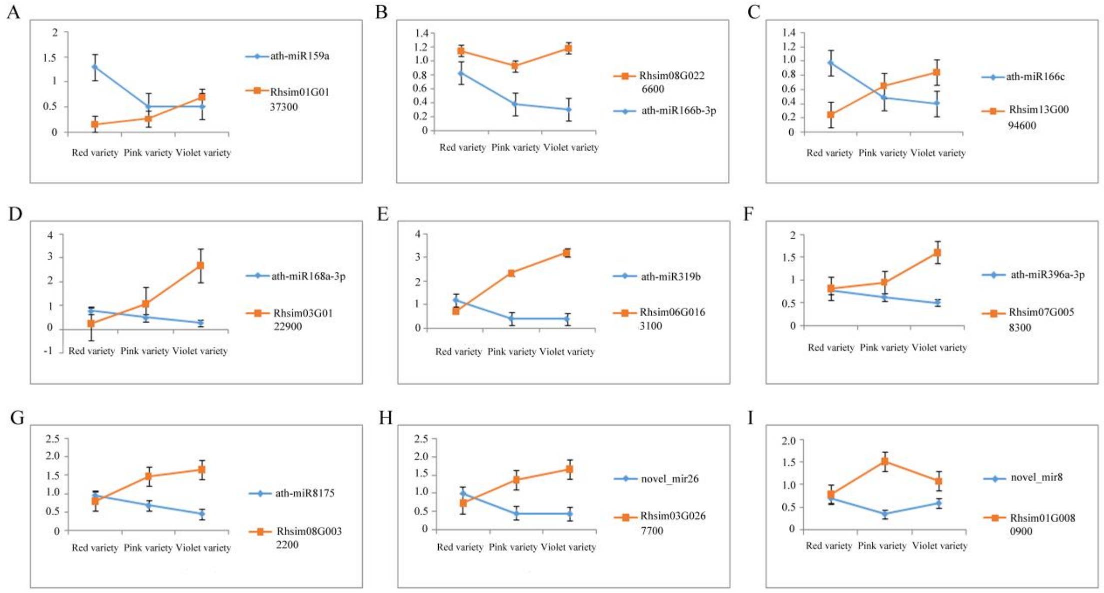

Figure S5 Expression analysis of eight selected miRNAs and corresponding target genes by real-time PCR.
